# Learning a Motor Skill from Video and Static Pictures in Physical Education Students—Effects on Technical Performances, Motivation and Cognitive Load

**DOI:** 10.3390/ijerph17239067

**Published:** 2020-12-04

**Authors:** Cyrine H’mida, Olivier Degrenne, Nafaa Souissi, Ghazi Rekik, Khaled Trabelsi, Mohamed Jarraya, Nicola Luigi Bragazzi, Aïmen Khacharem

**Affiliations:** 1 High Institute of Sport and Physical Education, University of Sfax, Sfax 3000, Tunisia; sirinehmida@hotmail.fr (C.H.); nafaa_souissi@hotmail.com (N.S.); trabelsikhaled@gmail.com (K.T.); jarrayam@yahoo.fr (M.J.); 2 Research Laboratory: Education, Motricity, Sport Health, EM2S, LR19JS01, High Institute of Sport and Physical Education of Sfax, University of Sfax, Sfax 3000, Tunisia; ghazi.rek@gmail.com; 3 UFR SESS-STAPS, Paris-East Créteil University, LIRTES (EA 7313), 94000 Créteil, France; olivierdegrenne@hotmail.com (O.D.); aimen.khacharem@gmail.com (A.K.); 4 Physical activity, Sport and health, UR18JS01, National Observatory of Sport, Tunis 1003, Tunisia; 5 School of Public Health, Department of Health Sciences (DISSAL), University of Genoa, 16132 Genoa, Italy; 6 Laboratory for Industrial and Applied Mathematics (LIAM), Department of Mathematics and Statistics, York University, Toronto, ON M3J 1P3, Canada; 7 DeVisu (EA 2445), Polytechnic University of Hauts-de-France, 59313 Valenciennes, France

**Keywords:** human movement, video, sequentiality, static pictures, learning

## Abstract

The purpose of the current study was to compare the effectiveness of a video and three different formats of static pictures (simultaneous-permanent pictures, sequential-transient pictures and sequential-permanent pictures) on the acquisition and retention of a complex judo skill in novice young adults. One hundred and thirty-three first-year students in the certificate in Physical Education (PE) were randomly assigned to either: a static-simultaneous-permanent pictures condition (*n* = 30), a static-sequential-transient pictures condition (*n* = 29), a static-sequential permanent pictures condition (*n* = 36) or a video condition (*n* = 38). They were instructed to observe and reproduce a complex judo technique (*Ippon-Seoi-Nage*) immediately after the learning phase (including a sequence of three trials—the acquisition phase) and after one week without observation (the retention phase). The results showed that the continuous video generated better learning performances than all static pictures formats. Moreover, it has been shown that sequential-permanent pictures presentation was more effective than static simultaneous-permanent pictures and sequential-transient pictures. In addition to the human movement effect, complementary explanations in terms of cognitive load theory, perceptual continuity, mental animation and intrinsic motivation are suggested. Implications of the results for the effective design of instructional materials within PE context are discussed.

## 1. Introduction

Computer-based multimedia instruction has become an integral part of the physical education (PE) learning environment [1,2,3,4] and of techniques learning in professional athletes [5,6]. In computer-based multimedia instruction, learners are exposed to material in verbal as well as visual format. Even though verbal presentation formats (e.g., oral explanations provided by teachers) have long dominated in PE, there is encouraging evidence that student performance can be enhanced by the addition of visual displays, such as static pictures and videos, which portray the key step of a dynamic event or procedure [7,8,9]. However, there is still a lack of investigation on how to design and present these visual displays, so that they are best suited to achieve learning goals. Therefore, in the present study, we explored the effectiveness of different visual supports (static and dynamic) to optimize the learning of a specific motor skill in a judo context.

The positive role and use of learning/repeating a movement from a screen picture in a classroom has been recently investigated and the studies showed that exposure to the movement tasks can be helpful for the attitudes of pupils towards physical activity [10,11,12].

In the PE context, dynamic visualizations such as videos are increasingly being employed to communicate tactical and motor skills that are difficult to verbalize. Studies assessing the instructional and cognitive benefits of videos show that they are more effective than static pictures, when content-to-be learnt involve acquisition of a human movement [13]. It has been shown that mirror neurons are responsible for our ability to engage in imitative learning [14]. The mirror neuron system is a neurological system located in the premotor cortex that activates when humans observe an action performed by another individual. This observation is supposed to cognitively prime the observer to perform the same action [14]. Therefore, a video showing a human movement automatically can activate the mirror neuron system which is supposed to reduce the difficulties associated with processing transient information (i.e., the human movement effect [15]). When Ayres et al. [16] assessed the impact of instructional videos on learning a specific motor skill (e.g., knot tying), they found that videos were more effective than static pictures. Other research, however, has shown that complex dynamic representations such as videos or animations are not necessarily more effective than static ones for learning [17]. This is because the information they convey involves nonhuman movement and thus requires extra resources to integrate them in memory [18].

Although static pictures do not move (contrary to videos) they can effectively represent change over time when they are designed to promote mental animation that is, the ability to infer the motion from the information given in the static displays [19]. One promising way of communicating information about movement in static visualizations is to present a series of phase pictures or “small multiples” [20], depicting the evolution of a procedure or a system at different phases. Several studies showed the effectiveness of multiple static pictures in communicating dynamic behaviors. In a mechanical domain, Hegarty et al. [21] examined people’s comprehension of a flushing cistern after they viewed three static pictures. Peoples who viewed these phase diagrams had better understanding of the mechanical system compared to those who viewed a single static picture or a dynamic presentation. In sport related-fields, Khacharem et al. [22] demonstrated that a series of multiple static pictures that represented key stages of a football game system produced more efficient learning outcomes (combination of recall and cognitive lead) compared to dynamic presentation in novice players.

In contrast to a global presentation in which multiple static pictures are presented simultaneously (i.e., next to each other), it is possible to optimize the presentation format by progressively presenting the pictures on-screen (i.e., one after the other). Several studies on sequential presentation of pictures provide support for this argument. For a task in which learners had to rearrange a random sequence of the eight images representing kangaroo jumping, a static-sequential presentation of the learning materials was superior in comparison to a static-simultaneous presentation [23]. For understanding a game system in football, sequential presentation (with tracing) was more beneficial than simultaneous presentation. However, other studies have shown that a simultaneous presentation outperforms a sequential presentation. For learning a mechanical system, Boucheix and Scheider [24] showed that static simultaneous presentation was better than static-sequential presentation. For a locomotion pattern classification task, Imhof et al. [25] indicated that learning from statics-simultaneous visualizations was more beneficial than learning from statics-sequential visualizations. To explain the contradictory findings Imhof et al. [26] stated that whereas static-sequential visualizations are more appropriate to depict the order of separate states or phases, static-simultaneous visualizations may be more appropriate for tasks involving continuous movements.

In previous research, the conditions of static sequential presentation were not always specified. Static pictures can be presented in two different formats. In the first format, a new static picture appears on the screen while the previous picture remains available [27]. For example, when a picture representing phase 2 of a system projects on the screen, the picture showing the phase 1 can still be seen and is thus permanently accessible. This presentation format may facilitate mental animation of a dynamic system because it allows visual comparisons between non-transient information. In the second format, a novel picture appears on the screen while the previous one disappears [24]. For example, when a picture representing the second phase of a system appears on the screen, the picture showing the first phase vanishes. This presentation format requires learners to carry out effective mental comparisons between transient information to understand the depicted system, that is keeping the current information (from picture 2) active in working memory to integrate it with the previous information (from picture 1). This format looks like a dynamic animation or a flipbook since the rapid succession of pictures—alteration of the previous picture by the newest one—may create the illusion of flowing movement. 

Based on the rich results obtained in learning from static pictures in different academic domains, the present study aimed to compare the effectiveness of a video and three presentation formats of static pictures (simultaneous-permanent pictures, sequential-transient pictures and sequential-permanent pictures) on the acquisition and retention of a complex judo skill in novice young adults. To our knowledge, such direct comparison has never before been conducted within a motor acquisition skill domain. Based on the existing literature, we predicted that the video would lead to superior learning outcomes compared to the three static pictures presentation in both immediate and delayed tests (Hypothesis 1). Moreover, we predicted that both static presentations with permanent pictures—sequential-permanent pictures (Hypothesis 2a) and simultaneous-permanent pictures (Hypothesis 2b)—will lead to superior learning performances compared to static presentation without permanent pictures—sequential-transient pictures. 

## 2. Materials and Methods

### 2.1. Participants and Design

A total of 181 students were assessed for eligibility of the inclusion criteria. The inclusion criteria were: (i) they are novice practitioners (i.e., without any prior knowledge of judo techniques or rules or other grappling sport (e.g., Jiu-Jitsu)) and (ii) they have normal or corrected to normal vision. Due to these criteria, 19 participants were excluded. Also, from the selected 162 participants, 29 students were excluded due to their absence in the retention phase. Therefore, the data recorded from 133 students (64 males and 69 females; 4 left-handed and 129 right-handed) in the first-year students in the certificate in PE were included in the analysis. The age of the participants ranged from 18 to 22 years (age: 19.6 ± 0.9 year). They received a throughout explanation of the research design, aims and benefits before they gave their written consent to participate in the project. The study was approved by the local ethics committee and was conducted according to the declaration of Helsinki. The participants have responded to a demographic survey about gender, age, vision level and previous knowledge in Judo. They participated to three learning lessons of the basics of Judo (e.g., the *Judogi*, the grip (i.e., *Kumikata*), the positions, the displacement and the fall techniques). Then, they were quasi-randomly assigned to either: a static-simultaneous-permanent pictures condition (*n* = 30), a static-sequential-transient pictures condition (*n* = 29), a static-sequential permanent pictures condition (*n* = 36) or a video condition (*n* = 38).

### 2.2. Apparatus and Task

The task consists of performing an *Ippon-Seoi-Nage*. This is a hand technique frequently used with novice practitioners in the leaning of Judo techniques. The performance of the *Ippon-Seoi-Nage* needs two students. The first student is called “*Tori*” and he/she will execute the technique. The second partner is called “*Uke*” and he/she will realize the fall after his/her projection by *Tori*. For all students, the same partner acting as *Uke* (i.e., the same gender as Tori: one male *Uke* and one female Uke) participated to the task (this will reduce the resistance to the throw). During *Ippon-Seoi-Nage* technique, *Tori* throw *Uke* to the floor over his/her shoulder. 

For the continuous video visualization, the performance of the *Ippon-Seoi-Nage* of the expert model using a right grip (*Kumikata*) was recorded using a video camera (Canon LEGRIA HF G25 (1920 × 1080 pixels, 25 frames per second)). The duration of the video was 12-s. Two experts in Judo choose eight 240 × 180 pixels screenshots from this video to prepare the static simultaneous permanent pictures, sequential transient pictures and sequential permanent pictures presentation formats. The experts were judo athletes for more than ten years and judo teachers and trainers for more than five year. So, they were familiarized with the evaluation and determination of the keys elements of a judo technique. These pictures were presented for 12-s duration using a two rows single Microsoft PowerPoint page for the static simultaneous permanent pictures, the sequential transient pictures and the sequential permanent pictures presentation formats. 

**Figure 1:**
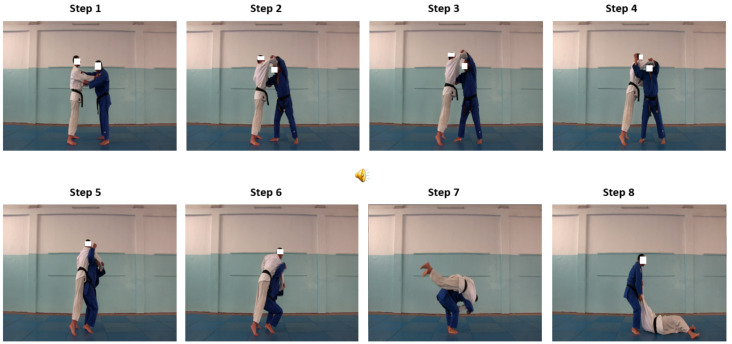
The static-simultaneous-permanent pictures presentation.

For the static simultaneous pictures, all images were presented at the same time and remain on the screen until the end of the 12-s (Figure 1). 

**Figure 2:**
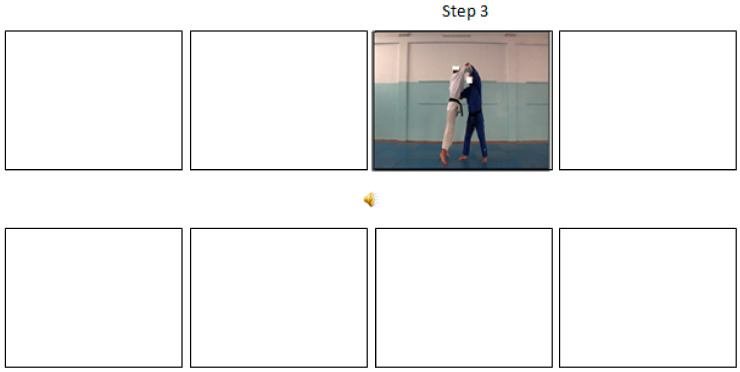
The sequential-static-transient pictures presentation (a screenshot from the Step 3 the judo movement).

For the sequential transient pictures, the images appear and disappear consecutively until the end of scrolling the eight pictures during 12-s. Figure 2 illustrate an example of this condition that is, when a picture representing the step 3 of the judo technique appears on the screen, the picture showing the step 2 vanishes from the screen.

**Figure 3:**
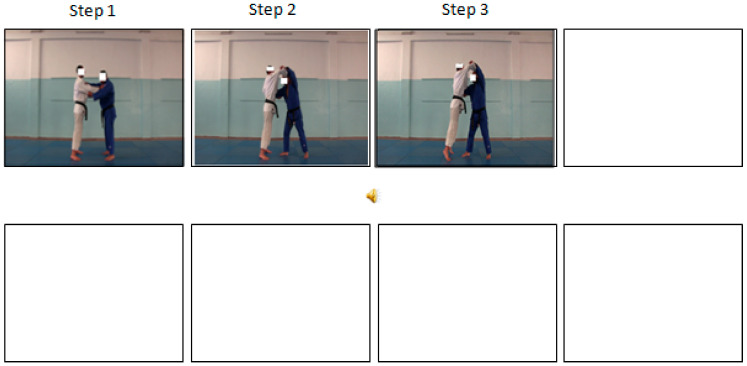
The sequential-static-permanent pictures presentation (a screenshot from the Step 3 of the judo movement).

For the sequential permanent pictures, the images appear and still consecutively until the end of the 12-s and the eight pictures. Figure 3 illustrate an example of this condition that is, when a picture representing step 3 of the judo technique projects on the screen, the picture showing the step 2 (and 1) remains available and is thus permanently accessible. 

All formats were presented using a 17-inch computer (i.e., Dell OptiPlex 7010) and a data show (Epson EB-S05) with a screen size of 1.4 × 1.0 m and at a height of 0.8 m. For all groups, the presentation was assisted by the same audio-recorded male voice explanation with neutral accent of the technique. At any time of the study and for all participants, no feedbacks or corrections were provided. Also, they were not allowed to see their performance of the technique at any time of the experiment. All the techniques realized during the two phases by each *Tori* were recorded using a tripod-mounted video camera (Canon LEGRIA HF G25 (1920 × 1080 pixels, 25 frames per second)) placed at 4 m from the student at a 1.5 m height.

### 2.3. Measures and Analysis

A total score ranging from “0” to “20” was calculated for each student according to ten fixed success criteria of the “*Ippon-Seoi-Nage*” that represent three technical phases (i) unbalancing *Uke* in the direction of the projection “*Tsukuri*,” (ii) perform a rotation for the placement of *Uke* in the back “*Kuzushi*” and (iii) throw *Uke* “*Kake*”) and was scored as 0 “absent,” 1 “imperfect execution” or 2 “correct execution” (Table 1). The two experts scored the recorded videos of the students. The video were watched by the experts independently and individually as many times as required and using slow motion if needed in random order. Also, they were blinded to the participant’ group. The video could be re-watched by the two experts together if a different score was recorded for the same student to obtain a final agreed score. 

For the acquisition phase, the total score of each participant was averaged by block to obtain three values. These values and the score of the retention phase were recorded for the statistical analysis.

### 2.4. Procedure

**Figure 4:**
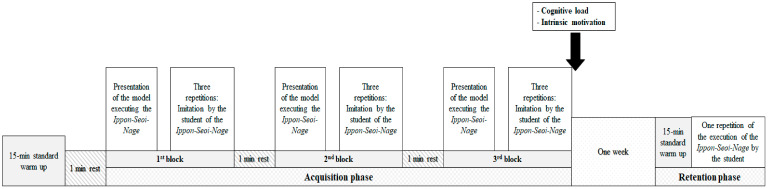
The experimental design of the study.

The experiment design is presented in Figure 4. The task and the procedure were carried out in a Judo hall and were the same for all participants of all groups and only the presentation formats differ. No participant has the opportunity to see the performance of another student and, so, the execution of the task was realized individually as *Tori*. The study consisted of two phases: an acquisition phase (during the 4th learning lesson of Judo) and a retention phase (during the 5th learning lesson of Judo). For the acquisition phase, the participants realized three blocks of three repetitions of the execution of the *Ippon-Seoi-Nage* after an observation of the model (i.e., static simultaneous permanent pictures, sequential transient pictures, sequential permanent pictures and continuous video) one time and after a 15 min standard warm up at the beginning of the session. A recovery period of one minute was given for all participants between the blocks. During each repetition, *Tori* was asked to project (*Nagekomi*) *Uke* on the *Tatami* while he imitates as closely as possible the technique of the model. The retention phase was realized after one week (without a prior knowledge of this phase) and consists of one block of one repetition of the *Ippon-Seoi-Nage* after a 15 min standard warm up without any presentation. During the 48 h preceding the acquisition and the retention, participants were invited to refrain from practicing physical exercises. 

At the end of the three blocks, the students’ perception of the cognitive load and the intrinsic motivation were calculated. As previously utilized by Khacharem [28], the cognitive load was calculated as the mean of the mental effort [29] and the perceived difficulty [30]. “How much mental effort did you invest to learn the task?” was the question utilized for the mental effort and “How difficult was it to learn the task?” was the question for the perceived difficulty. A nine points scale ranging from 1 “very very low” to 9 “very very high” was utilized for both questions. The intrinsic motivation was an adapted version of the Ryan’s [31] Intrinsic Motivation Inventory previously utilized by Badami et al. [32]. Nine questions were utilized to assess the interest/enjoyment, the perceived competence, the effort/importance and the pressure/tension. Responses to these questions ranged from 1 “strongly disagree” to 7 “strongly agree.” The Cronbach’s alphas were 0.90 for the interest/enjoyment, 0.88 for the perceived competence and 0.89 for the effort/importance.

### 2.5. Statistical Analysis

The STATISTICA 12.0 and Microsoft Excel 2010 (Microsoft Corp., Redmont, WA, USA) software were utilized for the statistical analysis of the data. The data were presented as mean ± standard error (SE) in the Figure and as mean ± standard deviation (SD) in the tables. The Kolmogorov-Smirnov test and the Mauchley test of the sphericity of the data confirmed the normality of the Gaussian distribution. Accordingly, a two-way [4 (Groups) × 4 (Phases)] analysis of variance (ANOVA) was utilized for the technical scores and a one-way ANOVA [4 (Groups)] was used for the cognitive load and the intrinsic motivation. Partial eta-squared values of 0.01, 0.06 and 0.13 represented small, moderate and large effect sizes, respectively [33]. When significant main effect or interaction was recorded, a pair-wise comparison was performed using the Bonferroni post-hoc test. Partial eta-squared (ɳ_p_^2^) was calculated as the effect size. Standardized effect size (Cohen’s *d*) analysis was used to interpret the magnitude of differences between variables and classified as follow: ≤0.20 (trivial); ≤0.60 (small); ≤1.20 (moderate); ≤2.0 (large); ≤4.0 (very large); >4.0 (extremely large) [33]. Significant main effect, interaction or pair-wise comparisons were considered when the alpha level was ≤0.05. Except when alpha level was <0.0005, exact *p* values were reported.

## 3. Results

### 3.1. Technical Scores

**Figure 5:**
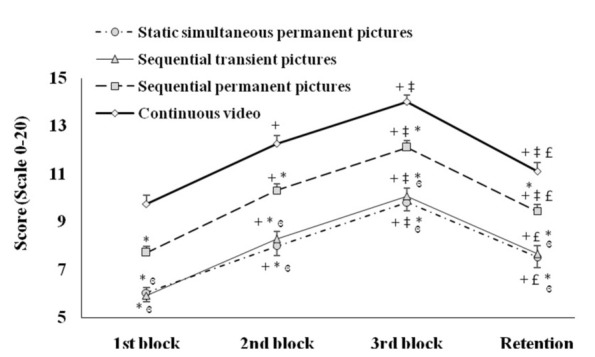
Technical scores (mean ± SE) recorded for the static simultaneous permanent pictures, the sequential transient pictures, the sequential permanent pictures and the continuous-video groups during the three blocks and the retention phase. +: Significant difference compared to the 1st block; ‡: Significant difference compared to 2nd block; £: Significant difference compared to 3rd block; ɞ: Significant difference compared to the sequential permanent pictures group; *: Significant difference compared to the continuous video.

The technical scores recorded for the static simultaneous permanent pictures, the sequential-transient pictures, the sequential permanent pictures and the continuous video groups during the three blocks and the retention phase are presented in Figure 5. 

The statistical analysis revealed significant main effects for Groups (F(3, 129) = 34.55; *p* < 0.0005; ɳ_p_^2^ = 0.44 (large)) and Phases (F(3, 129) = 745.87; *p* < 0.0005; ɳ_p_^2^ = 0.85 (large)) and a significant interaction Groups × Phases (F(9, 387) = 1.92; *p* = 0.0474; ɳ_p_^2^ = 0.04 (moderate)).

The post-hoc analysis indicated that technical scores were significantly higher in the continuous video group compared to the static simultaneous permanent pictures, the sequential-transient pictures and the sequential-permanent pictures groups during the 1st (*p* < 0.0005, *d* = 9.9 (extremely large), *p* < 0.0005, *d* = 11.1 (extremely large) and *p* = 0.0015, *d* = 6.5 (extremely large) respectively), the 2nd (*p* < 0.0005, *d* = 11.8 (extremely large), *p* < 0.0005, *d* = 12.1 (extremely large) and *p* = 0.0029, *d* = 6.5 (extremely large) respectively) and the 3rd (*p* < 0.0005, *d* = 13.1 (extremely large), *p* < 0.0005, *d* = 12.3 (extremely large) and *p* = 0.0050, *d* = 6.7 (extremely large) respectively) blocks and the retention phase (*p* < 0.0005, *d* = 8.9 (extremely large), *p* < 0.0005, *d* = 9.5 (extremely large) and *p* = 0.0398, *d* = 5.1 (extremely large) respectively). Also, technical scores were significantly higher in the sequential permanent pictures group compared to the static simultaneous permanent pictures and the sequential-transient pictures groups during the 1st (*p* = 0.0498, *d* = 5.0 (extremely large) and *p* = 0.0398, *d* = 6.0 (extremely large) respectively), the 2nd (*p* < 0.0005, *d* = 6.9 (extremely large) and *p* = 0.0057, *d* = 6.7 (extremely large) respectively) and the 3rd (*p* = 0.0005, *d* = 7.2 (extremely large) and *p* = 0.0061, *d* = 6.5 (extremely large) respectively) blocks and the retention phase (*p* = 0.0129, *d* = 5.3 (extremely large) and *p* = 0.0377, *d* = 5.5 (extremely large) respectively).

For the static simultaneous permanent pictures, the sequential-transient pictures, the sequential-permanent pictures and the continuous video groups, technical scores were lower (i) during the 1st block compared to the 2nd (*p* < 0.0005 for all, *d* = 5.0 (extremely large), *d* = 7.1 (extremely large), *d* = 9.8 (extremely large) and *d* = 8.5 (extremely large) respectively) and the 3rd (*p* < 0.0005 for all, *d* = 10.2 (extremely large), *d* = 12.4 (extremely large), *d* = 16.2 (extremely large) and *d* = 14.3 (extremely large) respectively) blocks and the retention phase (*p* < 0.0005 for all, *d* = 3.6 (very large), *d* = 5.0 (extremely large), *d* = 6.4 (extremely large) and *d* = 4.8 (extremely large) respectively), (ii) during the 2nd block compared to the 3rd block (*p* < 0.0005 for all, *d* = 4.9 (very large), *d* = 5.4 (extremely large), *d* = 6.5 (extremely large) and *d* = 5.6 (extremely large) respectively) and (iii) during the retention phase compared to the 3rd block (*p* < 0.0005, for all, *d* = 5.8 (very large), *d* = 6.9 (extremely large), *d* = 9.5 (extremely large) and *d* = 8.7 (extremely large) respectively). 

For the sequential permanent pictures and the continuous video groups, also, technical scores were lower during the retention phase compared to the 2nd block (*p* < 0.0005 for all, *d* = 3.2 (very large) and *d* = 3.3 (very large), respectively). 

No significant difference between the static simultaneous permanent pictures group and the sequential-transient pictures group was reported during the three blocks and the retention phases (*p* = 1.00).

### 3.2. Cognitive Load

The mental effort, the perceived difficulty and the cognitive load recorded for the static simultaneous permanent pictures, the sequential transient pictures, the sequential permanent pictures and the continuous video groups are presented in Table 2.

The statistical analysis revealed a significant main effect for Groups for the mental effort (F(3, 129) = 13.03; *p* < 0.0005; ɳ_p_^2^ = 0.23 (large)), the perceived difficulty (F(3, 129) = 13.67; *p* < 0.0005; ɳ_p_^2^ = 0.24 (large)) and the cognitive load (F(3, 129) = 14.68; *p* < 0.0005; ɳ_p_^2^ = 0.25 (large)).

The mental effort, the perceived difficulty and the cognitive load were significantly lower (*i*) for the continuous video group compared to the static simultaneous permanent pictures (*p* < 0.0005 for all, *d* = 1.2 (moderate), *d* = 1.3 (moderate) and *d* = 1.3 (moderate) respectively), the sequential transient pictures (*p* < 0.0005 for all, *d* = 1.3 (moderate), *d* = 1.2 (moderate) and *d* = 1.3 (moderate) respectively) and the sequential permanent pictures groups (*p* = 0.0287, *d* = 0.7 (moderate), *p* = 0.0466 *d* = 0.6 (small) and *p* = 0.0243, *d* = 0.7 (moderate) respectively) and (*ii*) for the sequential permanent pictures group compared to the static simultaneous permanent pictures (*p* = 0.0475, *d* = 0.7 (moderate), *p* = 0.0266, *d* = 0.8 (moderate) and *p* = 0.0354, *d* = 0.7 (moderate) respectively) and the sequential transient pictures groups (*p* = 0.0435, *d* = 0.7 (moderate), *p* = 0.0432, *d* = 0.7 (moderate) and *p* = 0.0399, *d* = 0.7 (moderate) respectively). However, no significant difference between the static simultaneous permanent pictures group and the sequential transient pictures group was observed.

### 3.3. Intrinsec Motivation

The scores of interest/enjoyment, perceived competence, effort/importance and intrinsic motivation recorded for the static simultaneous permanent pictures, the sequential transient pictures, the sequential permanent pictures and the continuous video groups are presented in the Table 3.

The statistical analysis revealed a significant main effect for Groups for the interest/enjoyment (F(3, 129) = 11.64; *p* < 0.0005; ɳ_p_^2^ = 0.21 (large)), the perceived competence (F(3, 129) = 11.47; *p* < 0.0005; ɳ_p_^2^ = 0.21 (large)), the effort/importance (F(3, 129) = 13.34; *p* < 0.0005; ɳ_p_^2^ = 0.23 (large)) and the intrinsic motivation (F(3, 129) = 12.61; *p* < 0.0005; ɳ_p_^2^ = 0.22 (large)).

The interest/enjoyment, perceived competence, effort/importance and intrinsic motivation were significantly higher (i) for the continuous video group compared to the static simultaneous permanent pictures (*p* < 0.0005 for all, *d* = 1.1 (moderate), *d* = 1.1 (moderate), *d* = 1.2 (moderate) and *d* = 1.2 (moderate) respectively), the sequential transient pictures (*p* < 0.0005 for all, *d* = 1.2 (moderate), *d* = 1.2 (moderate), *d* = 1.3 (moderate) and *d* = 1.3 (moderate) respectively) and the sequential permanent pictures groups (*p* = 0.0490, *d* = 0.5 (small), *p* = 0.0496, *d* = 0.5 (small), *p* = 0.0307, *d* = 0.6 (moderate) and *p* = 0.0492, *d* = 0.6 (moderate) respectively) and (ii) for the sequential permanent pictures group compared to the static simultaneous permanent pictures group (*p* = 0.0423, *d* = 0.7 (moderate), *p* = 0.0476, *d* = 06 (moderate), *p* = 0.0383, *d* = 0.7 (moderate) and *p* = 0.0377, *d* = 0.7 (moderate) respectively). However, no significant difference between the sequential transient pictures group and (i) the static simultaneous permanent pictures group and (ii) the sequential permanent pictures group was observed.

## 4. Discussion

The first hypothesis predicted that learners in the video group would outperform those in the three static groups. This prediction was confirmed on the motor task for both acquisition and retention tests. This performance advantage was obtained with lower cognitive load and higher intrinsic motivation. Indeed, the video group ranked their invested mental effort and their difficulty levels significantly lower than the static groups. These results showing a video advantage for learning of a human motor skill are in line with the research showing the superiority of videos over static presentation in learning of human motor skills [16,34,35]. The information transience effect generally associated to learning from continuous videos was not found because the activation of the mirror neuron system. Furthermore, the instructional video was found to be adapted to participants’ cognitive resources and was not too long or complex to become subject to negative effects of information transience. These findings are in contrast to some researches [24,25,26] reporting that dynamic visualizations do not outperform a static-simultaneous presentation of multiple pictures, which suggests that instructional superiority of videos is only limited to contents that involve acquisition of human motor skills [13]. Finally, it should be noted that superiority of instructional video over static pictures could be extended to an ecological (non-laboratory) learning task combining model observation and practice (i.e., re-enacting the movements that have just been observed). 

The second objective of the study is to determine whether permanence of static pictures was necessary to effectively integrate the judo technique. In agreement with hypothesis 2a, the permanent-sequential picture presentation, which allowed direct visual comparisons between different states of the movement, produced better performances than the sequential-transient-static picture presentation (i.e., learners attained higher technical performances in both acquisition and retention tests with a lower investment of mental effort and a higher intrinsic motivation). In the sequential-transient-static picture presentation each new image representing a new state of the movement caused the disappearance of the previous state before it is fully processed; as a consequence, the perceptual continuity of movement was disrupted and this created difficulties in retaining essential information for learning. According to cognitive load theory [36], which is concerned with how the design of instructions affects working memory and learning, this processing imposes a high working memory load that is likely to interfere with learning [37]. However, for permanent-sequential picture presentation, there are no such supplementary cognitive demands as the different states of the movement are permanently available to the learner and can be reviewed any required number of times. Also, in the present study, the same verbal instruction was given to all participants for all condition. Although this could supplement the visual representation, the addition of verbal instruction could increase the cognitive load [38].

Furthermore, contrary to our expectations (hypothesis 2b), the results showed that the simultaneous-permanent picture presentation generated low performance (in terms of learning scores and invested mental effort) compared to the sequential-permanent picture presentation. It is possible that this presentation format triggered a divided attention effect as learners were required to dissociate their visual attention between the different pictures—representing the key step of the judo technique—(from 1 to 8) and effectively integrate the whole movement. According to cognitive load theory [39], the divided-attention effect occurs when participants have to split their attention between multiple sources of information in order to mentally integrate a specific event. This effect is expected to amplify the unproductive cognitive load and cause negative effects in terms of construction and automation of knowledge. Moreover, it can be supposed that simultaneous presentation of multiple pictures may involve complex visual search to effectively locate and integrate the orally evoked information. These difficulties are avoided in the case of sequential-permanent presentation as the learner’s visual attention is automatically captured by the sudden appearance of each novel step/phase of the movement, alleviating therefore perceptual-cognitive demands and optimizing learning performances. 

The present research offers insight into the instructional effectiveness of static pictures and videos in learning a specific motor skill. However, an important factor that was not explored in this study is the effect of some moderator factors such age, gender and spatial ability on learning from the presentation formats. For example, evidence from an aptitude-treatment interaction perspective showed that instructional formats that are effective for low spatial ability learners may become ineffective for high spatial ability learners and vice versa [40,41]. Moreover, the study was based on a pure observational learning context. We recognize that incorporation of supplementary instructional designs such as segmentation and self-control can impact the learning performances. Finally, like all empirical investigations it is essential to reproduce the findings using different learning materials. 

## 5. Conclusions

It can be concluded that for this particular motor skill (*Ippon-Seoi-Nage*), a video instruction is more helpful for learners than equivalent static format. Furthermore, the results also showed that using a permanent-sequential-picture presentation is an interesting option of communicating a content that involve a human motor skill if there are no equipment and facilities to create or exhibit an instructional video (ex. if the PE teacher does not have such equipment). As we continue to explore how to optimize learning from dynamic and static presentations within PE context, it will be important to consider the characteristics of the participant, the learning objective and the pedagogical context into consideration. 

## Figures and Tables

**Table 1:** Different phases of the *Ippon-Seoi-Nage* and their related success criteria.

Phases	Criteria	Scores
Break of balance	1-Pull forward and up the right arm of *Uke*	0, 1 or 2
	2-Pull the left back of the *Judogi* of *Uke* by bending the right arm	0, 1 or 2
	3-Placement of the right arm under the armpit of *Uke*	0, 1 or 2
Placement	1-Complete the body rotation	0, 1 or 2
	2-Foot placement (i.e., spreading, bending, on the same line)	0, 1 or 2
	3-Contact between the back of *Tori* and the body of *Uke*	0, 1 or 2
Projection	1-The liberation of the armpit of *Uke* by the right arm	0, 1 or 2
	2-Direction of the projection of *Uke*	0, 1 or 2
	3-Maintaining balance by *Tori* during the projection phase	0, 1 or 2
	4-Continuity of the projection movement	0, 1 or 2

**Table 2:** Mental effort, perceived difficulty and cognitive load (mean ± SD) recorded for the static simultaneous permanent pictures, the sequential transient pictures, the sequential permanent pictures and the continuous video groups.

	Static Simultaneous Permanent Pictures	Sequential Transient Pictures	Sequential Permanent Pictures	Continuous Video
Mental Effort	4.80 ± 1.52 *^,ɞ^	4.86 ± 1.25 *^,ɞ^	3.94 ± 1.35 *	3.00 ± 1.51
Perceived Difficulty	4.90 ± 1.35 *^,ɞ^	4.83 ± 1.42 *^,ɞ^	3.92 ± 1.23 *	3.05 ± 1.49
Cognitive Load	4.85 ± 1.39 *^,ɞ^	4.84 ± 1.26 *^,ɞ^	3.93 ± 1.22 *	3.03 ± 1.42

ɞ: Significant difference compared to the sequential permanent pictures group; *: Significant difference compared to the continuous video group.

**Table 3:** Interest/enjoyment, perceived competence, effort/importance and intrinsic motivation (mean ± SD) recorded for the static simultaneous permanent pictures, the sequential transient pictures, the sequential permanent pictures and the continuous video groups.

	Static Simultaneous Permanent Pictures	Sequential Transient Pictures	Sequential Permanent Pictures	Continuous Video
Interest/enjoyment	2.69 ± 1.09 *^,ɞ^	2.78 ± 0.73 *	3.40 ± 1.00 *	4.01 ± 1.25
Perceived competence	2.71 ± 1.08 *^,ɞ^	2.75 ± 0.75 *	3.37 ± 0.95 *	3.94 ± 1.15
Effort/importance	2.71 ± 1.05 *^,ɞ^	2.79 ± 0.64 *	3.39 ± 0.97 *	4.04 ± 1.16
Intrinsic motivation	8.11 ± 3.2 *^,ɞ^	8.32 ± 2.04 *	10.16 ± 2.87 *	11.09 ± 3.46

ɞ: Significant difference compared to the sequential permanent pictures group; *: Significant difference compared to the continuous video group.

## References

[1] Leser R., Baca A., Uhlig J. (2001). Effectiveness of multimedia-supported education in practical sports courses. J. Sports Sci. Med..

[2] Mohnsen B. (2008). Using Technology in Physical Education.

[3] Lim W.Y., Koh M. (2006). Effectiveness of learning technologies in the teaching and learning of gymnastics. Pac. Asian Educ. J..

[4] Papastergiou M., Pollatou E., Theofylaktou I., Karadimou K. (2014). Examining the potential of web-based multimedia to support complex fine motor skill learning: An empirical study. Educ. Inform. Technol..

[5] Huang C., Zhang Y., Zhu C., Zhang C., Meng H. (2019). Chinese sports basketball teaching tactics training system combined with multimedia interactive model and virtual reality technology. Multimed. Tools Appl..

[6] Gunawan G., Firmansyah D., Widiastuti W. (2019). Effect of interactive multimedia learning to learn skills of students sports volleyball. J. Educ. Health Sport..

[7] Mayer R.E. (2009). Multimedia Learning.

[8] Schnotz W., Bannert M. (2003). Construction and interference in learning from multiple representation. Learn. Instr..

[9] Sweller J., Van Merrienboer J.J., Paas F.G. (1998). Cognitive architecture and instructional design. Educ. Psychol. Rev..

[10] Glapa A., Grzesiak J., Laudanska-Krzeminska I., Chin M.K., Edginton C.R., Mok M.M.C., Bronikowski M. (2018). The impact of brain breaks classroom-based physical activities on attitudes toward physical activity in polish school children in third to fifth grade. Int. J. Environ. Res. Public Health.

[11] Biljana P., Orce M., Katerina M.P., Snezana J.M. (2020). Different teaching strategies and methods applied at phe classes-experiences of classrom teachers. Res. Phys. Educ. Sport Health.

[12] Mok M.M.C., Chin M.K., Korcz A., Popeska B., Edginton C.R., Uzunoz F.S., Pasic M. (2020). Brain Breaks^®^ Physical Activity Solutions in the Classroom and on Attitudes toward Physical Activity: A Randomized Controlled Trial among Primary Students from Eight Countries. Int. J. Environ. Res. Public Health.

[13] Van Gog T., Paas F., Marcus N., Ayres P., Sweller J. (2009). The mirror neuron system and observational learning: Implications for the effectiveness of dynamic visualizations. Educ. Psychol. Rev..

[14] Rizzolatti G., Craighero L. (2004). The mirror-neuron system. Annu. Rev. Neurosci..

[15] Paas F., Sweller J. (2012). An evolutionary upgrade of cognitive load theory: Using the human motor system and collaboration to support the learning of complex cognitive tasks. Educ. Psychol. Rev..

[16] Ayres P., Marcus N., Chan C., Qian N. (2009). Learning hand manipulative tasks: When instructional animations are superior to equivalent static representations. Comput. Hum. Behav..

[17] Mayer R.E., Hegarty M., Mayer S., Campbell J. (2005). When static media promote active learning: Annotated illustrations versus narrated animations in multimedia instruction. J. Exp. Psychol..

[18] Bétrancourt M., Tversky B. (2000). Effect of computer animation on users’ performance: A review/(Effet de l’animation sur les performances des utilisateurs: Une sythèse). Le Travail Hum..

[19] Hegarty M. (1992). Mental animation: Inferring motion from static displays of mechanical systems. J. Exp. Psychol. Learn. Mem. Cogn..

[20] Tufte E.R. (1997). Visual Explanations: Images and Quantities, Evidence and Narrative.

[21] Hegarty M., Kriz S., Cate C. (2003). The roles of mental animations and external animations in understanding mechanical systems. Cogn. Instr..

[22] Khacharem A., Zoudji B., Ripoll H. (2013). Effect of presentation format and expertise on attacking-drill memorization in soccer. J. Appl. Sport Psychol..

[23] Lowee R.K., Schnotz W., Rasch T. (2010). Aligning affordances of graphics with learning task requirements. Appl. Cogn. Psychol..

[24] Boucheix J.M., Schneider E. (2009). Static and animated presentations in learning dynamic mechanical systems. Learn. Instr..

[25] Imhof B., Scheiter K., Gerjets P. (2011). Learning about locomotion patterns from visualizations: Effects of presentation format and realism. Comput. Educ..

[26] Imhof B., Scheiter K., Edelmann J., Gerjets P. (2012). How temporal and spatial aspects of presenting visualizations affect learning about locomotion patterns. Learn. Instr..

[27] Paas F., Van Gerven P.W., Wouters P. (2007). Instructional efficiency of animation: Effects of interactivity through mental reconstruction of static key frames. Appl. Cogn. Psychol..

[28] Khacharem A. (2017). Top-down and bottom-up guidance in comprehension of schematic football diagrams. J. Sports Sci..

[29] Paas F.G. (1992). Training strategies for attaining transfer of problem-solving skill in statistics: A cognitive-load approach. J. Educ. Psychol..

[30] Hasler B.S., Kersten B., Sweller J. (2007). Learner control, cognitive load and instructional animation. Appl. Cogn. Psychol..

[31] Ryan R.M. (1982). Control and information in the intrapersonal sphere: An extension of cognitive evaluation theory. J. Personal. Soc. Psychol..

[32] Badami R., VaezMousavi M., Wulf G., Namazizadeh M. (2011). Feedback after good versus poor trials affects intrinsic motivation. Res. Quart. Exerc. Sport.

[33] Lakens D. (2013). Calculating and reporting effect sizes to facilitate cumulative science: A practical primer for *t*-tests and ANOVAs. Front. Psychol..

[34] Höffler T.N., Leutner D. (2007). Instructional animation versus static pictures: A meta-analysis. Learn. Instr..

[35] Wong A., Marcus N., Ayres P., Smith L., Cooper G.A., Paas F., Sweller J. (2009). Instructional animations can be superior to statics when learning human motor skills. Comput. Hum. Behav..

[36] Sweller J., Ayres P., Kalyuga S., Sweller J., Ayres P., Kalyuga S. (2011). Measuring cognitive load. Cognitive Load Theory: Explorations in the Learning Sciences, Instructional Systems and Performance Technologies.

[37] Ayres P., Paas F. (2007). Making instructional animations more effective: A cognitive load approach. Appl. Cogn. Psychol..

[38] Kalyuga S. (2012). Instructional benefits of spoken words: A review of cognitive load factors. Educ. Res. Rev..

[39] Ayres P., Sweller J. (2005). The split-attention principle in multimedia learning. Camb. Handb. Multimed. Learn..

[40] Münzer S., Seufert T., Brünken R. (2009). Learning from multimedia presentations: Facilitation function of animations and spatial abilities. Learn. Individ. Differ..

[41] Höffler T.N. (2010). Spatial ability: Its influence on learning with visualizations—A metaanalytic review. Educ. Psychol. Rev..

